# Novel coronavirus and trauma surgery: successful infection control from a level I trauma centre

**DOI:** 10.1007/s00068-020-01435-9

**Published:** 2020-07-25

**Authors:** Péter Jávor, Endre Varga, Károly Fekete, Ferenc Tóth, Petra Hartmann

**Affiliations:** 1 Department of Traumatology, University of Szeged, Szeged, Hungary; 2 Department of Traumatology and Hand Surgery, University of Debrecen, Debrecen, Hungary; 3 Péterfy Hospital and Trauma Centre, Budapest, Hungary; 4 Institute of Surgical Research, University of Szeged, Pulz u. 1., Szeged, 6724 Hungary

**Keywords:** COVID-19, SARS-CoV2, Pandemic, Trauma, Patient care protocol

## Abstract

**Purpose:**

In the absence of effective treatment options, the recent SARS-CoV2 pandemic poses a great challenge to the health and social sectors worldwide. Hereby, we would like to share our proposals in the hope that it will prove helpful for our colleagues in this difficult time.

**Methods:**

The present recommendations are based on the opinion of experts as well as the experience of a group of traumatologists directly involved in the organization of traumatology wards. The reassignment of the healthcare personnel, the separation of the potentially infected patients and the different levels of restriction on the trauma care are all key elements of our protocol.

**Results:**

Since the first SARS-CoV2-positive case was confirmed in Hungary, our trauma surgeons were able to avoid contamination with the help of the new guidelines, without reducing the quality of trauma care.

**Conclusion:**

Reasonably adjusted patient care protocols in every medical field are key to contain the spread of infection and to avoid public health crisis. Sharing experience can be an important element of a successful fight against the recent pandemic.

**Electronic supplementary material:**

The online version of this article (10.1007/s00068-020-01435-9) contains supplementary material, which is available to authorized users.

## Introduction

In December 2019, multiple cases of atypical pneumonia of unknown etiology have occurred in Wuhan, a city of 11 million people in central China [1]. In January 2020, a novel coronavirus was identified as causative agent and named temporarily as 2019-new Coronavirus (2019-nCoV) [2]. Because of its close relation to Severe Acute Respiratory Syndrome Coronavirus (SARS-CoV) [3], 2019-nCoV was renamed to SARS-CoV2 on 11 February 2020 [4]. Because of its rapid global spread, Coronavirus Disease 2019 (COVID-19)—the disease caused by SARS-CoV2—was characterized as a pandemic by the World Health Organization (WHO) on the 11 march 2020 [5]. Until 8 April 2020, 1,353,361 confirmed cases and 79,235 deaths have been reported worldwide [6]. The increasing number of symptomatic cases, the rapid progression of respiratory symptoms in the susceptible population and the absence of effective treatment options have caused a public health crisis in multiple countries. Therefore, the development of efficient guidelines for the prevention of viral transmission has become extremely urgent. Statistics showing relatively high percentages of health care personnel among the infected increase the importance of infection control even further [7, 8]

The first confirmed SARS-CoV2-positive cases in Hungary were announced on the 4 March 2020. Since then, several outbreak control measures have been administered and the different fields of healthcare have been adjusted to the current situation nation-wide, resulting in a successful mitigation of the spread so far. The Traumatology Department of the University of Szeged is one of the leading level I trauma centers in the country, and the center of Advanced Trauma Life Support (ATLS) education in Hungary. In collaboration with the University of Debrecen, our institute has composed a multitude of patient management proposals and carried out significant changes to the in-hospital and outpatient trauma care. Until now, every trauma surgeon in our team has managed to avoid infection by complying strictly with our guidelines. Hereby, we would like to present our recommendations with respect to trauma care during COVID-19 pandemic. It evolves the reasonable assignment of the healthcare personnel, the earliest possible separation of the potentially or proven virus-infected patients and the different levels of restriction on the trauma care depending on the load on the care system. We truly believe that every successful infection control routine is worth sharing in the current situation.

## Reassignment of the staff

Despite of the strict preventive measures, every trauma unit has to be prepared for the possibility of a spreading infection among the personnel. Two or three separate medical teams must be formed according to the human resources and progressivity level of the individual hospital.

**Fig. 1:**
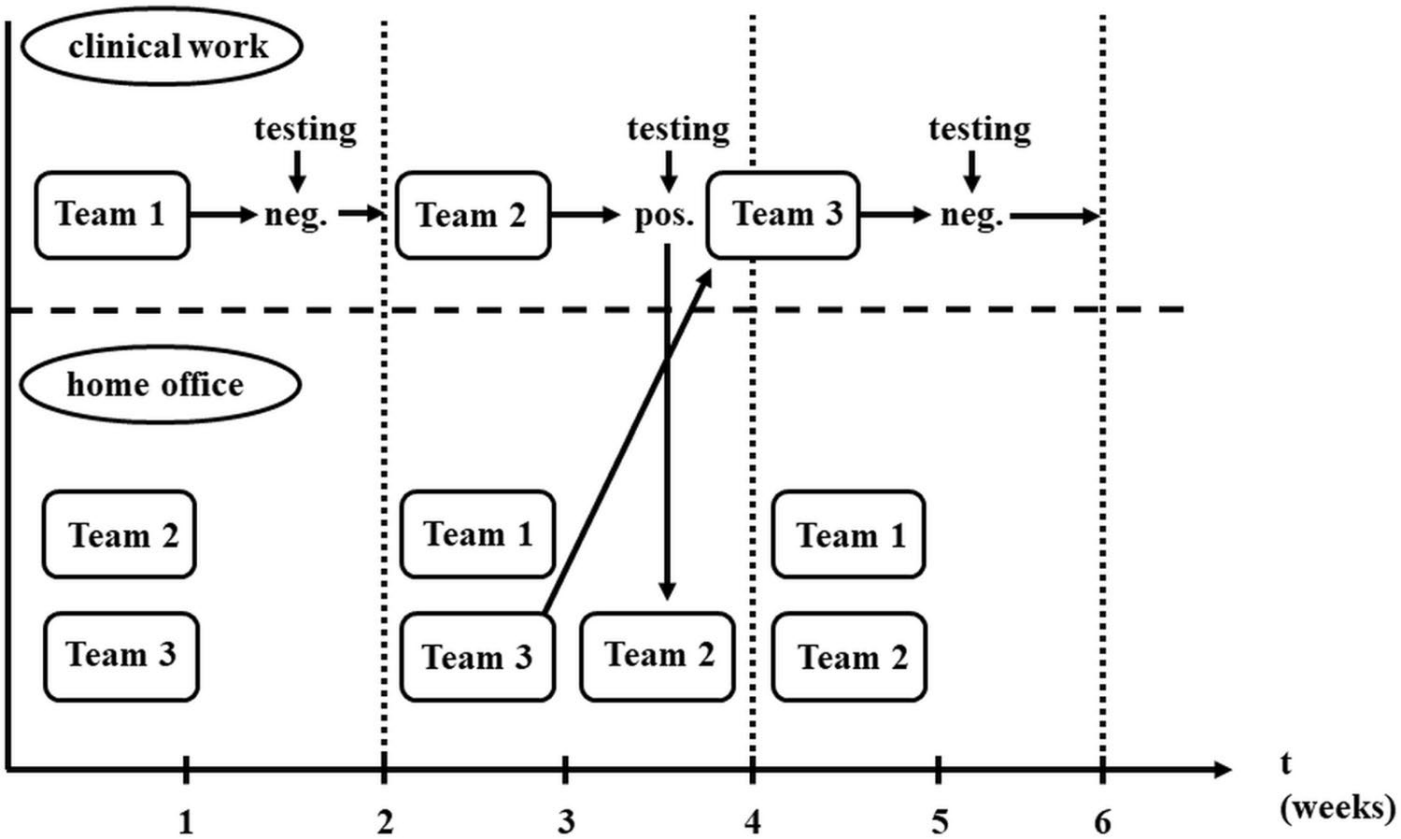
Illustration of rotational work schedule during SARS-CoV2 pandemic. Separate medical teams work in 2 week rotations. They get tested for SARS-CoV2 consistently. Only one team can work with patients at a time, in case of a contamination the team will be taken out from the clinical environment and replaced immediately by another team

Only one team can work with patients at a time, the others help with administrative and statistical duties, strictly in home isolation. In the absence of contamination, the teams work in a 2 week rotation schedule. A contamination has to be recognized as soon as possible. Therefore, the entire medical team must undergo diagnostic testing consistently. To be able to rule out infection with a high degree of certainty, two PCR tests with 1 week difference and an antibody test at the time of the second PCR need to be performed in our institute. In case of SARS-CoV2 positivity, the team must be isolated and replaced. The illustration of the rotational system is shown in Fig. 1.

Healthcare professionals over the age of 60 should be protected by being withdrawn from direct patient care. However, their clinical experience, routine and knowledge are required, highly appreciated and should be utilized by younger colleagues via regular online consultations.

To avoid unnecessary commuting, all inter-hospital residency rotations must be suspended temporarily, and all clinical residents should be recalled to their primary institutes.

## Infection control of patients

Each and every patient has to use the same, single entrance on arrival to the trauma ambulance, where they undergo a COVID-19 risk assessment which consists of a questionnaire and physical examination performed by medical professionals wearing appropriate protective equipment. Thereafter, patients are guided into separate parts of the trauma care unit based on their risk assessment results. Suspected SARS-CoV2 positive patients must undergo sampling for RT-PCR testing immediately. Until the arrival of results, they have to be isolated and treated as SARS-CoV2 positive. The contact personnel must be protected properly (special full cover clothing, FFP3 masks).

In the treatment of patients in extremis, risk assessment and diagnostic testing are delayed and SARS-CoV2 positivity is assumed automatically. To make such shortcuts possible, medical professionals have to be ready for the prompt management of potentially infected patients around-the-clock. Therefore, one trauma nurse dressed in personal protective equipment (PPE) and one trauma surgeon ready for the prompt donning of PPE are always available in our institute.

Modifications were made in several technical details of routine operative care also. In case there is a reasonable alternative, general anesthesia should be avoided. By airway generating procedures, only the required personnel in PPE are allowed to be present. Urgent, but not prompt emergency surgeries—such as pertrochanter- or femoral neck fractures—can be delayed by 24–48 h, till the result of diagnostic testing arrives. In case of a negative result and no need for airway manipulation, the operation can be performed under ordinary circumstances. Suspected or confirmed SARS-CoV2-positive patients can undergo surgery only in operating rooms that are designated for this purpose. OR personnel have to wear full PPE.

Regarding postoperative treatment, the early discharge of SARS-CoV2-negative trauma patients is a priority, even if it means that the principles of musculoskeletal rehabilitation have to be set aside temporarily. The postoperative management of people with positive or unknown status is performed by our delegated trauma surgeon residents on a designated ward of infectious diseases. Strict isolation is obligatory in case of an unknown viral status, while cohort isolation is allowed for confirmed SARS-CoV2-positive patients if the capacity level of the ward makes it necessary. Faculty from the home isolation team is always available for our resident doctors for online consultations.

Ultimately, the practical aspects of trauma care must always be adapted to the current epidemiological situation.

**Fig. 2:**
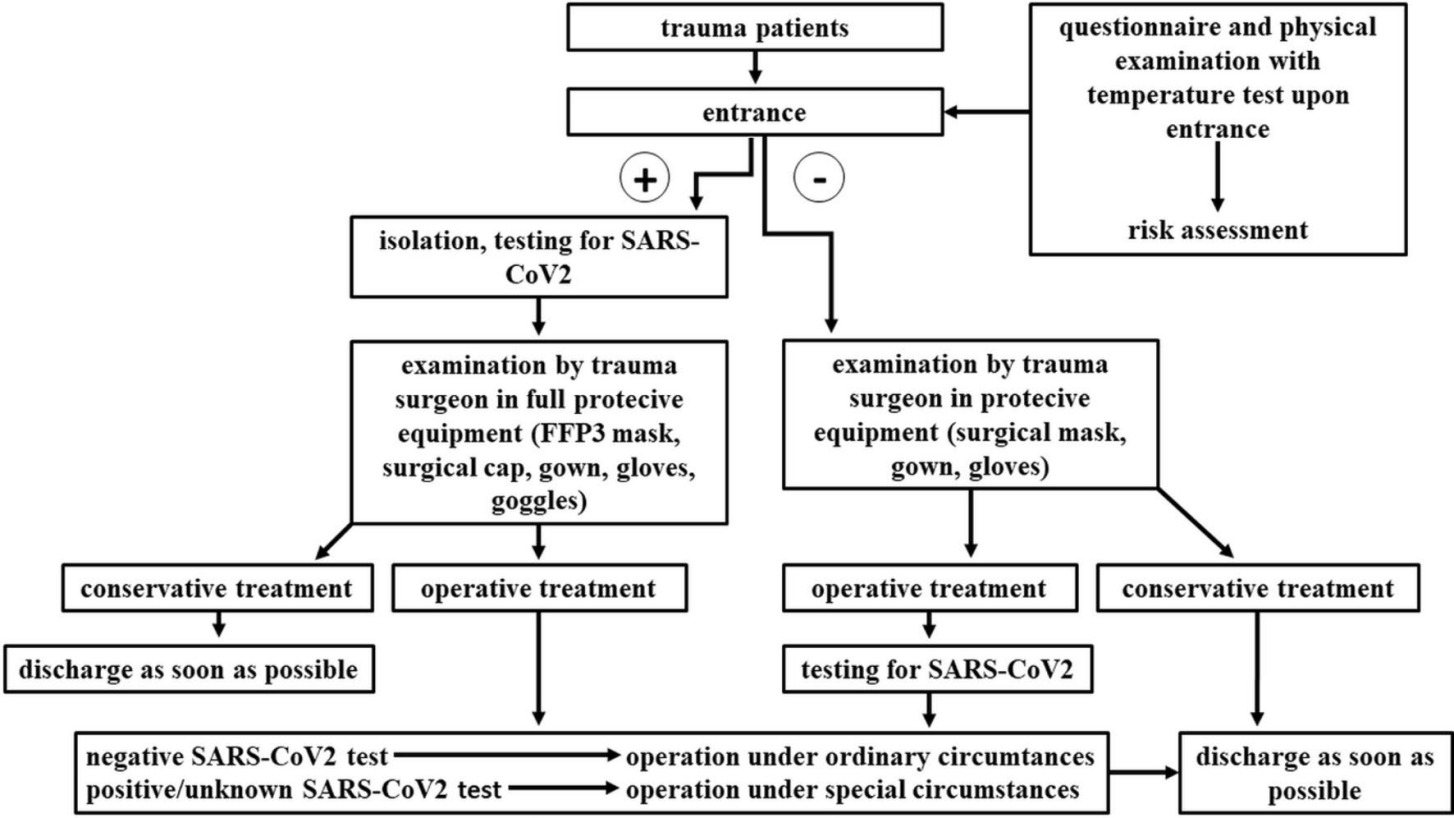
Trauma care protocol during SARS-CoV2 pandemic. The flowchart demonstrates our infection control system: first, all patents must undergo risk assessment. In case of a suspected infection, the patient has to be isolated and tested for SARS-CoV2 immediately. Every patient has to possess a test result before getting operative treatment, except for prompt emergency situations. In such cases, SARS-CoV2 positivity must be assumed. Our medical professionals wear protective equipment by every patient contact. We strive for the early discharge of our patients whenever it is possible

Our patient management protocol is demonstrated on Fig. 2.

## Restrictions in trauma care

Our experts elaborated two levels of trauma care restrictions, as follows:

1. First, the indications for operative treatment have been limited.To decrease the risk of contamination by reducing the interactions of patients to healthcare workers and to other patients, elective surgical procedures have been postponed or cancelled. Conservative treatment has become preferred in case of closed tendon- and peripheral nerve injuries and closed fractures that are eligible for closed reduction and conservative treatment without the risk of permanent functional impairment. Consequently, most extra-articular fractures without step deformities should not be operated. It has to be taken into consideration that by patients over 65 years of age, the risks of a SARS-CoV2 infection might exceed the consequences of an imperfectly healed fracture, thus conservative treatment can be an acceptable compromise even by dislocated and intra-articular fractures. Because of the high rates of potentially life-threating complications due to immobilisation, the hip fractures of the older generation constitute an exception. It has to be emphasized, that choosing the proper treatment method in trauma surgery is often a highly complex decision. The patient population shows a great diversity both in age and general health condition, and often the ones that are the most susceptible to a fulminant COVID-19 need a surgical treatment the most. Consequently, universal recommendations are difficult to formulate. Every case needs a unique cost–benefit evaluation and risk assessment.
2. The second level of restrictions becomes indicated by the insufficient capacity of anaesthesia care in case of a widespread outbreak. In such situation, only a very limited amount of conditions, such as injuries penetrating into body cavities, life threatening cranio-cerebral or spinal injuries, open fractures, profound bleeding or compartment syndrome constitute an indication for acute operative treatment. In less severe cases, only professional wound care is recommended. Conservative fracture management has to be preferred, even it entails the need for multiple correctional operations once the epidemiological situation subsides.

## Patient demographics in our study period

From 04 March 2020 to 11 April 2020, 3656 patients entered to our Emergency Department. 52.77% of all patients were female, while there was a slight male dominance (53.32%) in the number of the injured. Almost a thousand people [986, (26.97%)] received trauma care of which 276 were hospitalized. More than 20% of the hospitalized had an ISS > 15. As a consequence of the epidemiological situation, the number of road traffic accidents was relatively low. In our study period, the most common mechanisms of injury were falls, including ground level falls and falling from a height. Patient demographics and mechanisms of injury are demonstrated in Table 1.

**Table 1:** Demography of patients in our emergency department between 04 March 2020 and 11 April 2020 and mechanisms of injury in trauma care

Total number of cases	3656
Age median (IQR)	56.09 (39.68–75.69)
Male *n* (%)	1727 (47.23)
Cases with involvement of a trauma surgeon *n* (%)	**986** (26.97)
Age median (IQR)	48.38 (28.16–69.77)
Male *n* (%)	562 (53.34)
ISS median (IQR)	9 (3–16)
Admission to trauma ward *n* (%)	**276** (27.99)
ISS > 15 points; *n* (%)	**61** (22.10)
ISS median (IQR)	24 (17–26)
Mechanism of injury *n*	
Road traffic accidents	81
Pedestrian	8
Motorcycle	10
Automobile	22
Bicycle	41
Automobile	22
Falls	391
Assault	45
Autoaggression	10
Burns (electrical burns)	55 (4)
Crush injury	8
Cuts	57
Animal bites	30
Other	309

26.97% of the total number of emergency cases needed the involvement of a trauma surgeon. 22.10% of the hospitalized trauma patients had an ISS > 15. The most common mechanisms of injury were falls, including ground level falls and falling from a height

## Summary

The current epidemiological situation poses a great challenge to the entire health- and social care sector. Hard decisions have to be made and there might be no perfect options to choose. Nonetheless, the principle is unchanged: taking the interests and safety of our patients as first priority. We do hope that our colleagues can benefit from the recommendations above, and it may help to overcome this difficult time we are all facing now.

## Electronic supplementary material

Below is the link to the electronic supplementary material.

Supplementary file1 (DOCX 12 kb)
