# Supplementary material for: Novel coronavirus and trauma surgery: successful infection control from a level I trauma centre
Source: Eur J Trauma Emerg Surg. 2020 Jul 25;46(4):737–41. doi: 10.1007/s00068-020-01435-9 (PMC7382560; doi:10.1007/s00068-020-01435-9)
Supplement: Supplementary file 1 — Supplementary file1 (DOCX 12 kb) [file 68_2020_1435_MOESM1_ESM.docx]

*Supplementary appendix*

*“Novel coronavirus and Trauma Surgery – Successful infection control from a level I Trauma centre” by Jávor et al.*

*Supplementary table 1.* Assignment of trauma surgeons and anesthesiologist during SARS-CoV2 pandemic. Trauma surgeons have worked in 2-week rotations, only 50% have had patient contact at the same time, thus they could have been replaced immediately in case of contamination. Anesthesiologist have followed a different, but well structured schedule, where only 60% of the total team were allowed to have patient contact at the same time. The presented numbers include both faculty and resident doctors.

| Number of trauma surgeons in the „clinical team” | 13 |
| --- | --- |
| Number of trauma surgeons in the „home isolation team” | 13 |
| Number of trauma surgeons delegated to infectology ward | 2 |
| Number of anesthesiologists working with patients at the same time | 53 |
| Total number of anesthesiologists available | 97 |
